# PrEP implementation by local health departments in US cities and counties: Findings from a 2015 assessment of local health departments

**DOI:** 10.1371/journal.pone.0200338

**Published:** 2018-07-25

**Authors:** Gretchen Weiss, Dawn K. Smith, Sarah Newman, Jeffrey Wiener, Alyssa Kitlas, Karen W. Hoover

**Affiliations:** 1 National Association of County and City Health Officials, Washington, District of Columbia, United States of America; 2 Division of HIV/AIDS Prevention, National Center for HIV/AIDS, Viral Hepatitis, STD, and TB Prevention, Centers for Disease Control and Prevention, Atlanta, Georgia, United States of America; Asociacion Civil Impacta Salud y Educacion, PERU

## Abstract

**Objective:**

The United States Public Health Service released clinical practice guidelines for daily oral preexposure prophylaxis (PrEP) in May 2014. Local health departments (LHDs) are expected to play a critical role in PrEP implementation. We surveyed LHDs to assess awareness of and interest in supporting PrEP implementation, what roles they were taking, or believed they should take, in supporting PrEP, and what resources would be required to do so.

**Methods:**

LHDs were surveyed in 2015 to assess their engagement in PrEP implementation (n = 500). The study employed a cross-sectional survey design with a randomly selected stratified sample.

**Results:**

Among responding LHDs (n = 284), 109 (29%, weighted proportion) reported engagement in PrEP implementation. LHDs serving large jurisdictions (population 500,000+) and located in the West were more likely to be engaged in PrEP implementation. Making referrals for PrEP (74%) and conducting education and outreach to community members (51%) were the activities most frequently reported by LHDs engaged in PrEP implementation; 45% anticipated expanding their level of engagement. Among LHDs not engaged in PrEP implementation, 13% expected to become engaged over the next four years, 46% were undecided, and 41% reported it was unlikely. Information about PrEP for health care providers and information about PrEP for health department staff were the most frequently reported resource needs for LHDs engaged and not engaged in PrEP implementation, respectively.

**Conclusions:**

PrEP implementation by LHDs was limited in 2015, three years after Food and Drug Administration approval and one year after the U.S. Public Health Service issued clinical practice guidelines. PrEP is a recently available intervention that is requiring LHDs to adjust existing HIV prevention efforts and service delivery models. Additional resources and implementation research is needed to effectively support PrEP scale-up by LHDs. Efforts must also be undertaken to increase PrEP awareness, knowledge, and implementation capacity among LHDs.

## Introduction

Goal 1 of the National HIV/AIDS Strategy for the United States (U.S.): Updated to 2020 (NHAS 2020) is to reduce by 25% the number of new HIV infections diagnosed in the U.S. in 2020 compared to the number diagnosed in 2010 [1]. To achieve this goal, the NHAS 2020 includes a call for clinical and public health organizations to provide full access to daily oral antiretroviral preexposure prophylaxis (PrEP) services to those for whom it is appropriate.

It is now well established, based on the results from several large clinical trials and open label studies, that PrEP with daily doses of co-formulated tenofovir disoproxil fumarate (300 mg) and emtricitabine (200 mg)–marketed in the U.S. with the brand name Truvada by Gilead Sciences, Foster City, California–is safe and highly effective for HIV prevention. With high medication adherence, PrEP can reduce the risk of HIV acquisition by >90% among those with sexual exposure risk and >70% among those with injection risk. The U.S. Food and Drug Administration (FDA) has approved an HIV prevention indication for Truvada, and the Centers for Disease Control and Prevention (CDC) has published clinical practice guidelines for PrEP use, to reduce the risk for acquiring HIV infection among gay, bisexual and other men who have sex with men (MSM), heterosexually active women and men (HET), and persons who inject drugs (PWID) with specific behavioral and clinical indications [2].

CDC estimates that 1.2 million Americans without HIV infection currently engage in sexual or drug use behaviors that place them at ongoing substantial risk of acquiring HIV infection, and so would benefit from the use of PrEP to reduce that risk. This number includes approximately 1 in 4 MSM, 1 in 5 PWID, and 1 in 20 HET [3].

Following the first trial result in 2010, the FDA approval in 2012, and especially the CDC guidelines in 2014, increases in both clinician awareness and the number of PrEP prescriptions have been documented [4, 5]. However, a major barrier remains the low awareness of PrEP among persons who would benefit from its use [6–8]. In the past year or two, as state and federal funding has been provided to health departments for PrEP implementation, including public awareness campaigns, clinician education, and engagement of community-based organization (CBO) partners, a steep increase in prescriptions has been documented [9, 10].

Because significant PrEP implementation and scale-up only began in 2014, little is known about the ways in which key HIV program leaders will incorporate this clinical intervention into what has been an HIV prevention effort primarily focused on non-clinical risk reduction interventions, educational campaigns, and HIV testing [11, 12]. Local health departments (LHDs) are expected to be critical partners in raising awareness of PrEP among community members and health care providers. LHDs can also be key actors in making PrEP available to those who would benefit from its use as frequent providers of 1) HIV testing and counseling, a necessary first step in identifying HIV-uninfected persons with significant risk behaviors, 2) STD care and partner services for populations often with indications for PrEP use, and 3) funding services for key populations through CBOs. However, to do this effectively, LHDs will need to increase their own knowledge of PrEP [13] and determine their roles in PrEP delivery, based on their existing HIV and STD prevention services, resources and structure, and community needs.

We conducted a survey of LHDs to gain an understanding of: 1) their awareness of and interest in supporting PrEP as part of their HIV prevention activities; 2) what roles they were taking, or believed they should take, in supporting PrEP access for persons in local populations at substantial risk of HIV infection and: 3) what resources would be required to support local PrEP implementation.

## Methods

The study employed a cross-sectional survey design with a randomly selected stratified sample, aiming to assess the roles, activities, needs, and next steps for LHDs in supporting PrEP implementation in their communities.

### Data collection

We assessed LHD engagement in PrEP via a web-based survey of 500 LHDs. The survey sample was drawn from 1,433 LHDs which indicated that they provide or contract out HIV or STD screening and/or treatment in the National Association of County and City Health Officials’ (NACCHO’s) 2013 National Profile of Local Health Departments [14] survey. Of the 2,527 LHDs that received the Profile survey in 2013, 2,000 responded, and 1,433 indicated that they provide or contract out HIV or STD screening and/or treatment.

The sampling was stratified by U.S. census region (Midwest, Northeast, South, and West), and regions with high rates of new HIV diagnoses as reported in the 2011 HIV surveillance report [15], were oversampled. Within each region, the sampling was additionally stratified by the size of the jurisdiction served by the LHD (less than 50,000, 50,000–499,999, and 500,000 or more people). Since LHDs with large population sizes represent a relatively small proportion of all LHDs, those LHDs were also oversampled to ensure a sufficient number of responses from large LHDs for the analysis. The sampling process was designed to produce national estimates but not to produce state-level estimates. The survey was piloted by six LHDs recruited from NACCHO advisory groups. Based on feedback from respondents, final revisions to the survey were made.

NACCHO distributed the survey to 500 LHDs from July to September 2015 using Qualtrics, an online survey tool. The survey was e-mailed to the individual designated as best suited to respond to inquiries about PrEP (based on response to an initial e-mail sent by NACCHO to the LHDs in the survey sample), or, for those that did not respond to this initial inquiry, the health official for the LHD. After the initial e-mail invitation to the LHD PrEP contact, potential participants received up to four reminder emails. In addition, NACCHO made reminder calls to LHDs that had yet to complete the survey.

### Measures

The survey included three sets of questions. All surveyed LHDs received the first set of questions, assessing LHD HIV prevention program structure, services, and current engagement in PrEP. Engagement in PrEP was defined broadly to include any activities to address PrEP delivery (e.g., planning for incorporating PrEP into existing HIV prevention activities, conducting education and outreach, making referrals to PrEP, providing PrEP via a health department clinic). LHDs that indicated they were engaged in PrEP implementation received a second set of questions to further assess means of engagement in PrEP implementation, intentions to expand engagement in PrEP implementation, and resources needed to advance PrEP implementation. LHDs who indicated they were not engaged in PrEP implementation received an alternative second set of questions to assess PrEP awareness, knowledge, interest, potential future engagement in PrEP implementation, and resources needed to initiate PrEP implementation.

### Data analysis

Statistical analyses were conducted using Stata version 13.1 and SAS version 9.3. (Cary, NC) Sampling weights were computed for each stratum (by U.S. Census region and size of jurisdiction served) and analyses were weighted to account for the stratified survey design. Descriptive statistics and weighted proportions were calculated for all LHDs responding to the survey and by LHD characteristics including engagement in PrEP (yes; no). LHD characteristics were compared by engagement in PrEP using Rao-Scott chi-square tests. All survey responses were self-reported by an LHD representative; NACCHO did not independently verify the data provided by LHDs.

The survey was determined not to require Institutional Review Board human subject review by the CDC, as it constituted a public health program needs assessment and evaluation activity. This determination was made by the Office of the Associate Director for Science in the National Center for HIV/AIDS, Viral Hepatitis, STD, and TB Prevention under criteria established by the CDC Human Subjects Protection Office. It was conducted under Office of Management and Budget No. 0920–0879.

## Results

A total of 284 LHDs (57%) responded to the first set of survey questions (Table 1) and varying numbers responded to either of the second sets of questions (Tables 2–4). LHDs from all population sizes served, and U.S. Census Regions, responded to the survey. After weighting responses, a large majority of LHDs reported direct provision of services associated with PrEP implementation, including HIV screening/testing (84%), STD screening (81%), STD partner services (80%), and operating an STD clinic (65%) (Table 1).

**Table 1:** Characteristics of local health departments responding to PrEP implementation survey, United States, 2015, weighted response proportions.

Characteristic	Engaged in PrEP	Not Engaged in PrEP	p-value
	**(n = 109)**	**(n = 175)**	
**All Local Health Departments**	29%	71%	
**Population Size Served**			< 0.001
Small (less than 50,000)	18%	82%	
Medium (50,000–499,999)	32%	68%	
Large (500,000 or more)	68%	32%	
**Census Region**			0.001
Midwest	16%	84%	
Northeast	37%	63%	
South	33%	67%	
West	47%	53%	
**Direct Provision of HIV/STD Services**^**a**^			
HIV testing	98%	78%	< 0.001
HIV behavioral prevention counseling and/or interventions	91%	60%	< 0.001
HIV treatment	23%	7%	< 0.001
HIV partner services	77%	26%	< 0.001
HIV linkage to care	83%	38%	< 0.001
STD screening	88%	78%	0.10
STD prevention counseling and interventions	95%	83%	0.002
STD partner services	86%	77%	0.22
**Operate an STD Clinic(s)**^**b**^			
Yes	81%	58%	0.001

PrEP, preexposure prophylaxis; STD, sexually transmitted disease.

^a^n = 275–283

^b^n = 282

**Table 2:** Local health department PrEP implementation activities, optimal roles, and potential future roles, United States, 2015, weighted response proportions.

Implementation Activities and Roles	Engaged in PrEP: Activities	Engaged in PrEP: Optimal Roles^a^	Not Engaged in PrEP: Potential Future Roles
	(n = 108)	(n = 108)	(n = 173)
Education and outreach to community members	51%	58%	48%
Education and outreach to health care providers	40%	54%	31%
Internal training for health department staff	36%	40%	40%
Convene or participate in a working group on PrEP	32%	31%	19%
Develop local PrEP provider directories	45%	59%	53%
Referral to PrEP	74%	76%	74%
Delivery of PrEP from a health department clinic	9%	27%	15%
Collaborate with health care providers to support PrEP delivery	45%	55%	38%
Fund CBOs and other agencies to implement PrEP	3%	6%	5%
Monitor and evaluate PrEP use and impact	9%	23%	9%
Participation in demonstration project or implementation study	5%	14%	11%
Other	6%	1%	0%
Same as current activities	-	16%	-
None of the above	-	-	8%

PrEP, preexposure prophylaxis; CBO, community-based organization.

^a^ Local health departments engaged in PrEP implementation were asked what they see as their optimal role as it relates to implementing PrEP in their jurisdiction, given current or realistic resources.

**Table 3:** Challenges to PrEP implementation for local health departments, United States, 2015, weighted response proportions.

Challenges	Engaged in PrEP	Not Engaged in PrEP
	(n = 106)	(n = 174)
Lack of PrEP awareness and knowledge among staff	47%	23%
Lack of support from health department leadership	15%	6%
Uncertainty about effectiveness of PrEP for HIV prevention	22%	6%
Limited staff capacity to support PrEP implementation activities	61%	27%
Lack of health care providers willing to deliver PrEP	46%	9%
Concern about financial access to PrEP (for interested individuals)	53%	17%
Concern about inadequate reimbursement from third-party payers	27%	11%
Not sure what the health department should or could do	29%	18%
No significant challenges ^a^	17%	52%

PrEP, preexposure prophylaxis.

^a^ Answer choice: PrEP has not been an area of focus for our health department, so we have not faced any significant challenges to date

**Table 4:** Local health department expectation of expanding or initiating PrEP implementation, United States, 2015, weighted response proportions.

Expectation of Expansion or Initiation	Engaged in PrEP	Not Engaged in PrEP
	(n = 109)	(n = 174)
**All Respondents**^**a**^	45%	13%
**Population Size Served**		
Small (less than 50,000)	22%	8%
Medium (50,000–499,999)	42%	17%
Large (500,000 or more)	80%	48%
**Census Region**		
Midwest	48%	6%
Northeast	37%	3%
South	34%	22%
West	66%	24%

^a^ 41% of respondents engaged in PrEP and 46% of respondents not engaged in PrEP indicated they were not sure of their expectation to expand or initiate PrEP implementation.

### Engagement in PrEP implementation

Among 284 responding LHDs, 109 (29%, weighted proportion) reported engagement in PrEP implementation. LHDs serving large jurisdictions (population of 500,000+) and located in the West U.S. Census Region were more likely to be engaged in PrEP than those serving smaller populations (68% vs. 25%, p<0.001) and located in other regions (47% vs. 26%, p = 0.001). LHDs engaged in PrEP implementation were more likely to directly provide HIV and STD services associated with PrEP implementation than those not engaged, including HIV screening/testing (98% vs. 78%, p< 0.001), HIV treatment (23% vs 7%, p<0.001), HIV partner services (77% vs 26%, p<0.001), HIV linkage to care (83% vs 38%, p<0.001), and STD (95% vs 83%, p = 0.002) or HIV (91% vs 60%, p<0.001) prevention counseling and interventions, and were more likely to operate an STD clinic (81% vs. 58%, p = 0.001) (Table 1).

Making referrals to PrEP providers (74%), conducting PrEP education and outreach to community members (51%), developing local PrEP provider directories (45%), collaborating with healthcare providers to support PrEP delivery (45%), conducting PrEP education and outreach to health care providers (40%), conducting PrEP training events for LHD staff (36%), and convening or participating in a local or state working group on PrEP (32%) were the activities LHDs most frequently reported being engaged in for PrEP implementation (Table 2). Less frequently reported were monitoring and evaluating PrEP uptake and impact (9%), delivering PrEP via a health department clinic (9%), participating in a demonstration project or implementation pilot study (5%), and funding CBOs or other local agencies to support PrEP implementation (3%) (Table 2).

While 61% of LHDs who engaged in PrEP implementation received some local, state, or federal funding to support their efforts, 38% reported no specific funding to support PrEP implementation efforts. The most frequently reported challenges to incorporating PrEP implementation into the LHD’s HIV prevention efforts were limited staff capacity (61%), concerns about financial access to PrEP for interested individuals (53%), lack of PrEP awareness and knowledge among staff (47%), and lack of health care providers willing to prescribe PrEP (46%). Nearly one-third (29%) noted the challenge of not being sure what the health department should or could do to support PrEP implementation (Table 3).

### Awareness, knowledge, and interest among non-implementers

Among LHDs not engaged in PrEP implementation, there was very limited awareness and knowledge of PrEP. Twenty-three percent reported that LHD staff were not aware or knowledgeable about PrEP, but only 6% reported that LHD leadership was not supportive of incorporating PrEP into HIV prevention efforts. LHDs not engaged in PrEP also reported having very limited discussion of PrEP with other LHD staff (6%), HIV prevention partners (4%), and local health care providers (4%) within the past 12 months, and only 1% reported having met with or conducted assessments among community members or clinic patients to determine awareness, knowledge, and interest in PrEP. When asked if they thought PrEP had the potential to make a significant impact on reducing new HIV infections in their jurisdiction taking local incidence and prevalence into consideration, 25% agreed and 48% were unsure.

We asked LHDs not currently engaged in PrEP implementation what challenges they either have faced or anticipate facing for PrEP implementation. Just over half (52%) responded that since PrEP has not been an area of focus for the health department, they had not faced any significant challenges. Among those who have faced challenges or anticipate facing challenges, the most frequently reported were limited staff capacity (27%) and lack of PrEP awareness and knowledge among staff (23%). Nearly one in five (18%) noted the challenge of not being sure what the health department should or could do to support PrEP implementation (Table 3).

### Intentions to expand/initiate implementation, optimal roles, and resource needs

LHDs were asked about their intention to expand or initiate PrEP implementation. Among LHDs already engaged in PrEP implementation, 45% anticipated expanding their level of engagement, 41% were not sure, and 14% did not anticipate expanding their engagement in PrEP implementation (Table 4). LHDs in the West and Midwest and those serving large jurisdictions were more likely to report intention to expand their engagement than those in the South and Northeast and those serving medium and small jurisdictions. Among LHDs that reported not being engaged in PrEP implementation, 13% expected to become engaged over the next four years, 46% were undecided, and 41% reported it was unlikely that they would implement PrEP. LHDs in the West and South were more likely to report intention to initiate PrEP implementation than LHDs in the Midwest and Northeast. Similar to LHDs engaged in PrEP, large jurisdictions not engaged in PrEP were more likely to report intention to initiate than those serving medium and small jurisdictions. LHDs that agreed with the statement that PrEP had the potential to make a significant impact on reducing new infections in their jurisdiction were much more likely to indicate that they anticipate initiating PrEP than those that did not agree with the statement (32% vs. 7%).

We asked LHDs what they saw as their optimal role in PrEP implementation (for those already implementing) and what roles they could or would play in the future (for those not implementing). The three most frequently reported roles were the same for both groups of LHDs: referring high-risk individuals to PrEP (76% among engaged vs. 74% among not engaged), developing local PrEP provider directories (59% vs. 53%), and conducting community education and outreach (58% vs. 48%). Regarding PrEP delivery via health department clinics, 27% of LHDs engaged in PrEP implementation identified it as an optimal role, and only 15% of those not engaged identified it as a potential future role (Table 2).

When asked about what resources would be most helpful for advancing engagement in PrEP implementation, LHDs already implementing PrEP most frequently reported information about PrEP for health care providers (59%), protocols for PrEP referral from a health department clinic (41%), additional funding to support implementation (39%), education and outreach materials for community members (35%), and guidance or direction from the state health department (33%) (Table 5). LHDs that selected the need for additional funding were asked what they would do with additional funding. The most frequently reported uses were expanding health care provider education (61%), paying for program staff to conduct non-clinical PrEP services (47%), and evaluating PrEP uptake and impact (45%).

**Table 5:** Top five resources selected by local health departments as being most helpful for advancing or initiating PrEP implementation, United States, 2015, weighted response proportions.

Engaged in PrEP (n = 108)	%	Not Engaged in PrEP (n = 167)	%
Information about PrEP to share with health care providers	59	Information about PrEP for health department staff	75
Protocols for PrEP referral from a health department clinic	41	Additional funding	51
Additional funding	39	Guidance or direction from the state health department	46
Education and outreach materials for community members	35	Information about PrEP to share with health care providers	40
Guidance or direction from the state health department	33	Protocols for PrEP referral from a health department clinic	37

LHDs not engaged in PrEP implementation were asked what resources would be most helpful if they were to begin considering how to incorporate PrEP into HIV prevention education and services. The most frequently reported resource needs were information about PrEP for health department staff (75%), additional funding (51%), guidance or direction from the state health department (46%), information about PrEP to share with health care providers (40%), and protocols for PrEP referral from a health department clinic (37%) (Table 5). Overwhelmingly, the most frequently reported use of funding for PrEP implementation would be to plan for how to incorporate PrEP into HIV prevention education and services (69%).

## Discussion

In their capacity as key providers or funders of HIV prevention services, LHD engagement in PrEP implementation is critical for the scale-up of PrEP. This survey provides important information about the levels of LHD engagement in PrEP implementation in late 2015 and their anticipated level of engagement over the next few years. It also provides insight into what LHDs foresee as their optimal roles in PrEP implementation, and what resources are needed for supporting the achievement of these optimal roles.

Survey findings indicate that PrEP implementation by LHDs was limited in 2015, three years after FDA approval of PrEP and one year after CDC issued clinical practice guidelines. After weighting was applied, among responding LHDs, only 29% were engaged in PrEP implementation, and among the 71% of LHDs not engaged in PrEP implementation, only 13% expected to become engaged over the next four years. This presents an important opportunity for intervention with LHDs. The need to increase PrEP education for, and build PrEP implementation capacity among, LHDs is similar to findings from other surveys and studies about PrEP knowledge, interest, and roles among CBOs and health care providers [4, 11].

For both LHDs engaged in PrEP implementation and those not, there was significant alignment among what LHDs were doing to support PrEP implementation and what they saw as their optimal or potential future role in PrEP implementation. In all three categories (see Table 2), making referrals to PrEP was the most frequently reported means of engagement in PrEP implementation. Further, the magnitude of responses across all three categories was nearly the same (74–76%) and significantly greater than the next most frequently reported activities or role. It is also notable that many of the other most frequently reported roles that ranked highly are supportive of the referral process, including developing PrEP provider directories, educating health care providers, and conducting community education and outreach.

These findings point to a clearly recognized role for LHDs in building the infrastructure, systems, and policies for identifying and connecting individuals at substantial risk of acquiring HIV infection to local PrEP providers. This role is consistent with the 10 Essential Public Health Services [16], a useful framework for thinking about the role of LHDs across the full spectrum of PrEP delivery, or “PrEP cascade.” Though the referral role clearly was the predominant activity, this survey shows that LHDs were engaged in a wide variety of PrEP implementation activities.

Only 16% of LHDs engaged in PrEP implementation indicated that in 2015 they were already serving in what they perceive as their optimal role, and nearly half of LHDs not engaged in PrEP implementation said they were undecided about whether they would initiate PrEP implementation activities. This points to the need for additional research, information, and resources to support increases in LHD implementation of PrEP. LHDs reported that they needed information and strategies for increasing local provider capacity for PrEP delivery and establishing provider directories and protocols for successful referral models. Additionally, LHDs identified additional funding for PrEP implementation and guidance from their state health department among their top resource needs. This is vital, as LHD HIV prevention programs are primarily supported by HIV prevention funding provided by their state health departments.

If, as expected, state and federal funding for PrEP implementation increases over the next few years, the distribution of these resources must be carefully considered so as not to exacerbate existing disparities in PrEP knowledge, access, and uptake. Our survey findings indicate differences in engagement in PrEP implementation and intentions to expand or initiate PrEP implementation by region and population size served. HIV is not evenly distributed across the country and the focus of new resources should be areas where diagnosis rates are highest, such as the South and Northeast.

Our study has a few limitations. 43% of the sample did not respond to the survey, which may limit our ability to generalize findings to all LHDs that provide HIV or STD screening and/or treatment. At the time the survey was administered, there was limited funding available specifically designated for PrEP implementation. Since the survey data were collected, the federal government, as well as many state and local governments, have designated funding for PrEP implementation [17–19]. As such, our findings might not be representative of the current percentage of LHDs engaged in PrEP implementation.

## Conclusion

LHDs are on the front lines of public health, and many are leading the way and significantly contributing to local and state efforts to end the HIV epidemic [20–22]. Scale-up of PrEP, as well as other biomedical prevention interventions (i.e., post-exposure prophylaxis and treatment as prevention), are critical to the success of these efforts. PrEP is a recently introduced intervention, and one that will require many implementers to think about, and act differently in, their HIV prevention efforts. While knowledge gaps and limited resources present challenges to implementation scale-up, many LHDs have served as local PrEP champions and demonstrated commitment to expanding efforts to make PrEP more available to those who would benefit from its use.

Additional implementation research is needed to increase our knowledge about how LHDs can implement PrEP and most effectively support scale-up, taking into consideration variations in services (non-clinical and clinical) provided by LHDs, the health care systems and community contexts LHDs operate within, and funding and resources available to LHDs for PrEP-related activities. Further, we must continue to monitor and evaluate LHD engagement in PrEP implementation. As the delivery systems for PrEP expand and evolve, it is crucial that we continue to increase our understanding of the critical role of LHDs in PrEP implementation, and effectively respond by creating and adapting resources and strategies to support and promote LHD efforts to increase access to PrEP.

## Supporting information

S1 File Survey instrument. (PDF)

S2 File Survey codebook. (PDF)

S1 Dataset De-identified data set. (CSV)
