# Supplementary material for: PrEP implementation by local health departments in US cities and counties: Findings from a 2015 assessment of local health departments
Source: PLoS One. 2018 Jul 25;13(7):e0200338. doi: 10.1371/journal.pone.0200338 (PMC6059435; doi:10.1371/journal.pone.0200338)
Supplement: S2 File — (PDF) [file pone.0200338.s002.pdf]

PrEP for HIV Prevention Survey

Section I: HIV Prevention Program Structure, Services, and Engagement in PrEP Implementation

1. Are your health department's HIV and STD programs combined or separate? **(s1q1)**
   - ☐ **[1]** Combined
   - ☐ **[2]** Separate
   - ☐ **[3]** Other

2. Does your health department operate any STD clinics? **(s1q2)**
   - ☐ **[1]** Yes
   - ☐ **[0]** No → If checked, skip questions 2a-e

   2a. Does the STD clinic(s) currently prescribe PrEP? **(s1q2a)**
   - ☐ **[1]** Yes
   - ☐ **[0]** No

   2b. Does the clinic(s) have a place (e.g., clinical provider, community health center) to which you refer patients to PrEP? **(s1q2b)**
   - ☐ **[1]** Yes
   - ☐ **[0]** No

   2c. Does your clinic have sufficiently trained personnel to assist with the following aspects of PrEP?

	Yes	No	Don't know
Screening for indications for PrEP **(s1q2c_a)**	**[1]**	**[0]**	**[2]**
Prescribing PrEP and medical follow-up **(s1q2c_b)**	**[1]**	**[0]**	**[2]**
Assissting with insurance/payment for PrEP access **(s1q2c_c)**	**[1]**	**[0]**	**[2]**
Counseling on adherence **(s1q2c_d)**	**[1]**	**[0]**	**[2]**
Counseling on sexual and/or injection risk **(s1q2c_e)**	**[1]**	**[0]**	**[2]**

   2d. Does the clinic(s) currently prescribe nonoccupational post-exposure prophylaxis (nPEP)? **(s1q2d)**
   - ☐ **[1]** Yes
   - ☐ **[0]** No

   2e. Does the clinic(s) have a place to which you refer patients for nPEP? **(s1q2e)**
   - ☐ **[1]** Yes
   - ☐ **[0]** No

3. Please indicate how your health department provides the following services: (Select all that apply)

(Variable values: unchecked=0, checked=1)

	Provided directly	Contracted out	Provided by others in the community independent of	Not available in the community	Don't know

			health department funding		
HIV screening/testing	**(s1q3_1a)**	**(s1q3_1b)**	**(s1q3_1c)**	**(s1q3_1d)**	**(s1q3_1e)**
HIV behavioral prevention counseling and/or interventions	**(s1q3_2a)**	**(s1q3_2b)**	**(s1q3_2c)**	**(s1q3_2d)**	**(s1q3_2e)**
HIV treatment	**(s1q3_3a)**	**(s1q3_3b)**	**(s1q3_3c)**	**(s1q3_3d)**	**(s1q3_3e)**
HIV partner services	**(s1q3_4a)**	**(s1q3_4b)**	**(s1q3_4c)**	**(s1q3_4d)**	**(s1q3_4e)**
HIV linkage to care services	**(s1q3_5a)**	**(s1q3_5b)**	**(s1q3_5c)**	**(s1q3_5d)**	**(s1q3_5e)**
STD screening	**(s1q3_6a)**	**(s1q3_6b)**	**(s1q3_6c)**	**(s1q3_6d)**	**(s1q3_6e)**
STD prevention counseling and/or interventions	**(s1q3_7a)**	**(s1q3_7b)**	**(s1q3_7c)**	**(s1q3_7d)**	**(s1q3_7e)**
STD partner services	**(s1q3_8a)**	**(s1q3_8b)**	**(s1q3_8c)**	**(s1q3_8d)**	**(s1q3_8e)**

For LHDs that indicated they provide HIV screening/testing directly or contracted out **(s1q3_1a, s1q3_1b)**

3a. Does your health department offer rapid HIV testing? **(s1q3a)**

- ☐ **[1]** Yes
- ☐ **[0]** No → Skip questions 3b-d
- ☐ **[2]** Not at this time, but we are planning to in the next 6-12 months → Skip questions 3b-d

3b. Approximately what percentage of the HIV rapid testing is done with the following processes: (Please provide a number from 0 to 100)

Oral rapid: ___**(s1q3b_a)**___
Fingerstick rapid using an antibody test only: ___**(s1q3b_b)**___
Fingerstick rapid using an antigen/antibody test (i.e., 4th generation test): ___**(s1q3b_c)**___

3c. Does your health department offer viral load testing? **(s1q3c)**

- ☐ **[1]** Yes
- ☐ **[0]** No
- ☐ **[2]** Not at this time, but we are planning to in the next 6-12 months

3d. Does your health department screen for acute HIV infection (i.e., identify individuals with very recent HIV infection, including those who may test negative on antibody tests alone)? **(s1q3d)**

- ☐ **[1]** Yes, with 4th generation antigen/antibody tests
- ☐ **[2]** Yes, with viral load NAT testing
- ☐ **[3]** Yes, with **both** 4th generation antigen/antibody tests and viral load NAT testing
- ☐ **[0]** No
- ☐ **[4]** Not at this time, but we are planning to in the next 6-12 months

4. Does your health department bill public or commercial insurance directly for HIV and/or STD services provided by the health department? (Note: Selection of commercial insurance does not indicate you bill all commercial insurance companies, rather that you bill at least one commercial insurance company.) **(s1q4)**

- ☐ **[1]** Yes, public insurance (i.e., Medicaid and Medicare) only
- ☐ **[2]** Yes, commercial insurance only
- ☐ **[3]** Yes, public and commercial insurance
- ☐ **[0]** No, health department clinics do not currently bill

- ☐ **[4]** Not applicable/health department does not offer clinical services

5. Does Medicaid in your state currently pay for PrEP? **(s1q5)**

- ☐ **[1]** Yes
- ☐ **[0]** No
- ☐ **[2]** Don't know

6. Does Medicaid in your state currently pay for nPEP? **(s1q6)**

- ☐ **[1]** Yes
- ☐ **[0]** No
- ☐ **[2]** Don't know

7. HIV prevention, care, and treatment increasingly require collaboration between public health and healthcare. On a scale from 1 to 5, 1 being "Never collaborates" to 5 being "Always collaborates," how would you rate the extent of collaboration that exists between your health department and the healthcare system in your community (as it relates to HIV prevention, care, and treatment)? **(s1q7)**

1- Never collaborates	2	3	4	5-Always collaborates
**[1]**	**[2]**	**[3]**	**[4]**	**[5]**

8. Does your health department's HIV and/or STD program provide education/training, technical assistance, or quality improvement support to healthcare providers in the community? **(s1q8)**

- ☐ **[1]** Yes → Skip question 8b
- ☐ **[0]** No → Skip question 8a

8a. What methods does your health department use for conducting provider education/training? (Select all that apply) **(Variable values: unchecked=0, checked=1)**

- ☐ **(s1q8a_a)** Developing and disseminating print or electronic letters/bulletins with health information
- ☐ **(s1q8a_b)** Holding in-person training events with CMEs
- ☐ **(s1q8a_c)** Holding in-person training events without CMEs
- ☐ **(s1q8a_d)** Conducting Web-based educational events (e.g., webinars)
- ☐ **(s1q8a_e)** Public health/academic detailing
- ☐ **(s1q8a_f)** Promoting or supporting federal training center opportunities (e.g., AIDS Education and Training Center, STD/HIV Prevention Training Center)
- ☐ **(s1q8a_g)** Promoting or supporting CDC-funded HIV capacity building assistance opportunities
- ☐ **(s1q8a_h)** Other (please specify): ________**(s1q8a_htext)**________

8b. If your health department decided to begin providing education/training to healthcare providers in the community, which strategies would be most feasible? (Select all that apply) **(Variable values: unchecked=0, checked=1)**

- ☐ **(s1q8b_a)** Developing and disseminating print or electronic letters/bulletins with health information
- ☐ **(s1q8b_b)** Holding in-person training events with CMEs
- ☐ **(s1q8b_c)** Holding in-person training events without CMEs
- ☐ **(s1q8b_d)** Conducting Web-based educational events (e.g., webinars)
- ☐ **(s1q8b_e)** Public health/academic detailing
- ☐ **(s1q8b_f)** Promoting or supporting federal training center opportunities (e.g., AIDS Education and Training Center, STD/HIV Prevention Training Center)

- ◻ **(s1q8b_g)** Promoting or supporting CDC-funded HIV capacity building assistance opportunities
- ◻ **(s1q8b_h)** Other (please specify): _______**(s1q8b_htext)**_______
- ◻ **(s1q8b_i)** None of the above

9. Is your health department currently engaged in PrEP, in any manner (e.g., planning for incorporating PrEP into existing HIV prevention activities, conducting education and outreach, making referrals to PrEP, providing PrEP via a health department clinic)? **(s1q9)**

- ◻ **[1]** Yes
- ◻ **[0]** No → Skip to Section IIN

Section IIY: Health Department Engagement in PrEP Implementation

Sections IIY, IIIY, IVY are for those who are currently engaged in PrEP in any manner (checked yes to s1q9)

1. How is your health department engaged in PrEP? (Select all that apply) **(Variable values: unchecked=0, checked=1)**

- ☐ **(s2yq1_a)** Conducting community education and outreach
- ☐ **(s2yq1_b)** Conducting healthcare provider education and outreach
- ☐ **(s2yq1_c)** Conducting training events for health department staff
- ☐ **(s2yq1_d)** Convening or participating in a local or state working group on PrEP
- ☐ **(s2yq1_e)** Developing a list of local healthcare providers prescribing or willing to prescribe PrEP
- ☐ **(s2yq1_f)** Referring high-risk individuals to PrEP providers/programs
- ☐ **(s2yq1_g)** Delivering/prescribing PrEP via a health department clinic (e.g., STD clinic)
- ☐ **(s2yq1_h)** Collaborating with healthcare providers to support PrEP delivery (e.g., health department provides HIV/STD screening and/or adherence and behavioral risk-reduction counseling)
- ☐ **(s2yq1_i)** Funding community-based organizations and agencies to support PrEP implementation
- ☐ **(s2yq1_j)** Monitoring and evaluating PrEP uptake and impact
- ☐ **(s2yq1_k)** Participating in a demonstration site project/implementation pilot study
- ☐ **(s2yq1_l)** Other (please specify): ________**(s2yq1_ltext)**_______

2. How are your health department's PrEP activities funded/supported? (Select all that apply) **(Variable values: unchecked=0, checked=1)**

- ☐ **(s2yq2_a)** Local health department funding
- ☐ **(s2yq2_b)** State health department HIV funding (including indirect CDC funding)
- ☐ **(s2yq2_c)** General state health department funding (funding from the state not designated to a specific program)
- ☐ **(s2yq2_d)** Direct CDC funding
- ☐ **(s2yq2_e)** Grant from a pharmaceutical company
- ☐ **(s2yq2_f)** Foundation grant
- ☐ **(s2yq2_g)** Other (please specify): _______**(s2yq2_gtext)**_______
- ☐ **(s2yq2_h)** Unfunded/there is no specific funding to support this work
- ☐ **(s2yq2_i)** Don't know

3. Who are/were your health department's key partners for: (Select all that apply)

3a. **Planning** for the implementation of your health department's PrEP activities? **(Variable values: unchecked=0, checked=1)**

- ☐ **(s2yq3a_a)** Local CBO or ASO
- ☐ **(s2yq3a_b)** Community health center
- ☐ **(s2yq3a_c)** Hospital or other medical practice
- ☐ **(s2yq3a_d)** Local clinical "champion" for PrEP
- ☐ **(s2yq3a_e)** Local HIV Planning Group
- ☐ **(s2yq3a_f)** State HIV Planning Group
- ☐ **(s2yq3a_g)** Community members
- ☐ **(s2yq3a_h)** Academic institution
- ☐ **(s2yq3a_i)** State health department
- ☐ **(s2yq3a_j)** CDC
- ☐ **(s2yq3a_k)** National nongovernmental organization

- ☐ **(s2yq3a_l)** Industry/pharmaceutical company
- ☐ **(s2yq3a_m)** Another jurisdiction(s) already implementing PrEP
- ☐ **(s2yq3a_n)** Other (please specify): ______**(s2yq3a_ntext)**______
- ☐ **(s2yq3a_o)** None of the above

3b. **Implementing** your health department's PrEP activities? **(Variable values: unchecked=0, checked=1)**

- ☐ **(s2yq3b_a)** Local CBO or ASO
- ☐ **(s2yq3b_b)** Community health center
- ☐ **(s2yq3b_c)** Hospital or other medical practice
- ☐ **(s2yq3b_d)** Local clinical "champion" for PrEP
- ☐ **(s2yq3b_e)** Local HIV Planning Group
- ☐ **(s2yq3b_f)** State HIV Planning Group
- ☐ **(s2yq3b_g)** Community members
- ☐ **(s2yq3b_h)** Academic institution
- ☐ **(s2yq3b_i)** State health department
- ☐ **(s2yq3b_j)** CDC
- ☐ **(s2yq3b_k)** National nongovernmental organization
- ☐ **(s2yq3b_l)** Industry/pharmaceutical company
- ☐ **(s2yq3b_m)** Other (please specify): ______**(s2yq3b_mtext)**______
- ☐ **(s2yq3b_n)** None of the above

4. What factors influenced the decision to incorporate PrEP activities into your health department's HIV prevention activities? (Select all that apply) **(Variable values: unchecked=0, checked=1)**

- ☐ **(s2yq4_a)** Results from clinical trials demonstrating the efficacy of PrEP
- ☐ **(s2yq4_b)** FDA approval of Truvada for PrEP in July 2012
- ☐ **(s2yq4_c)** Release of the US Public Health Service/CDC Clinical Practice Guidelines for PrEP in May 2014
- ☐ **(s2yq4_d)** Information from other jurisdictions (i.e., having examples of what other health departments were doing to support PrEP)
- ☐ **(s2yq4_e)** Internal health department support for PrEP
- ☐ **(s2yq4_f)** Encouragement or direction from the state health department
- ☐ **(s2yq4_g)** Demand from the community
- ☐ **(s2yq4_h)** Availability of funding to support PrEP
- ☐ **(s2yq4_i)** Other (please specify): ______**(s2yq4_itext)**______

5. What challenges did your health department face to incorporating PrEP activities into its HIV prevention services and activities? (Select all that apply) **(Variable values: unchecked=0, checked=1)**

- ☐ **(s2yq5_a)** Lack of PrEP awareness and knowledge among health department staff
- ☐ **(s2yq5_b)** Lack of support or interest from health department leadership
- ☐ **(s2yq5_c)** Uncertainty about PrEP as an effective HIV prevention strategy (e.g., belief that the benefits are outweighed by possible behavioral disinhibition and increased STDs, or that PrEP is a party drug)
- ☐ **(s2yq5_d)** Limited staff capacity to support PrEP implementation activities
- ☐ **(s2yq5_e)** Lack of enough healthcare providers willing to provide PrEP
- ☐ **(s2yq5_f)** Concern about financial access to PrEP (for individuals interested in taking PrEP)
- ☐ **(s2yq5_g)** Concern about inadequate reimbursement from third-party payers for PrEP-related services
- ☐ **(s2yq5_h)** Health department was not sure what it should or could be doing
- ☐ **(s2yq5_i)** Other (please specify): ______**(s2yq5_itext)**______

- ☐ **(s2yq5_j)** The health department **did not** face any significant challenges → Skip question 5a

5a. Of the challenges you selected, which do you think was the biggest challenge? (Select only one) **(s2yq5a)**

- ☐ **[1]** Lack of PrEP awareness and knowledge among health department staff
- ☐ **[2]** Lack of support or interest from health department leadership
- ☐ **[3]** Uncertainty about PrEP as an effective HIV prevention strategy (e.g., belief that the benefits are outweighed by possible behavioral disinhibition and increased STDs, or that PrEP is a party drug)
- ☐ **[4]** Limited staff capacity to support PrEP implementation activities
- ☐ **[5]** Lack of enough healthcare providers willing to provide PrEP
- ☐ **[6]** Concern about financial access to PrEP
- ☐ **[7]** Concern about inadequate reimbursement from third-party payers for PrEP-related services
- ☐ **[8]** Health department was not sure what it should or could be doing
- ☐ **[9]** Other (Refers to the other specified in s2yq5_i)

6. Who in your health department is most likely to have a conversation about PrEP with a potential PrEP user? (Select up to three) **(Variable values: unchecked=0, checked=1)**

- ☐ **(s2yq6_a)** Sexual health/STD clinician
- ☐ **(s2yq6_b)** HIV clinician
- ☐ **(s2yq6_c)** Family planning practitioner
- ☐ **(s2yq6_d)** Disease Intervention Specialist (DIS)
- ☐ **(s2yq6_e)** Linkage to care specialist
- ☐ **(s2yq6_f)** HIV case manager
- ☐ **(s2yq6_g)** Outreach and education staff
- ☐ **(s2yq6_h)** Behavioral health specialist
- ☐ **(s2yq6_i)** Other (please specify): ________**(s2yq6_itext)**________

7. Has your health department determined a method for assessing how many individuals living in your jurisdiction are at substantial risk for HIV and might benefit from PrEP? **(s2yq7)**

- ☐ **[1]** Yes
- ☐ **[0]** No
- ☐ **[2]** Not yet, but we are engaged in conversation about how to do so

8. Is there a particular population(s) your health department is targeting its PrEP activities to? If yes, please select the target population(s). If no, please select "not applicable." **(Variable values: unchecked=0, checked=1)**

- ☐ **(s2yq8_a)** Men who have sex with men
- ☐ **(s2yq8_b)** Transgender
- ☐ **(s2yq8_c)** High-risk heterosexual women
- ☐ **(s2yq8_d)** High-risk heterosexual men
- ☐ **(s2yq8_e)** Injection drug users
- ☐ **(s2yq8_f)** HIV-discordant couples
- ☐ **(s2yq8_g)** Racial and ethnic minorities
- ☐ **(s2yq8_h)** Those recently diagnosed with syphilis or rectal gonorrhea or chlamydia
- ☐ **(s2yq8_i)** Other (please specify): ________**(s2yq8_itext)**________
- ☐ **(s2yq8_j)** Not applicable → skip question 8a

8a. What strategies has your health department employed to target PrEP to those most in need? (Select all that apply)

- ◻ **(s2yq8a_a)** Conducting focus groups with individuals from high-risk populations to inform messaging to the target population(s)
- ◻ **(s2yq8a_b)** Featuring images of individuals representing the target population(s) in education and outreach materials
- ◻ **(s2yq8a_c)** Collaborating with organizations and other groups representing the needs of the target population(s)
- ◻ **(s2yq8a_d)** Developing protocols and criteria for PrEP delivery and/or referral based on demographic and risk characteristics of the target population(s)
- ◻ **(s2yq8a_e)** Other (please specify): ______**(s2yq8a_etext)**______
- ◻ **(s2yq8a_f)** None of the above

9. How will your health department attempt to monitor the number of people on PrEP in your jurisdiction? (Select all that apply)
- ◻ **(s2yq9_a)** Use of data from healthcare provider electronic medical records (EMRs)
- ◻ **(s2yq9_b)** Use of health department clinic EMRs/medical records
- ◻ **(s2yq9_c)** Use of pharmacy databases
- ◻ **(s2yq9_d)** Use of insurance billing data (e.g., all-payer claims database)
- ◻ **(s2yq9_e)** Use of data from community surveys (e.g., local National HIV Behavioral Surveillance)
- ◻ **(s2yq9_f)** Use of data from local provider surveys
- ◻ **(s2yq9_g)** Other (please specify): ______**(s2yq9_gtext)**______
- ◻ **(s2yq9_h)** Don't know yet
- ◻ **(s2yq9_i)** This is not something the health department is considering monitoring at this time

10. Does your health department have a plan for how it will evaluate the impact of PrEP on HIV incidence in your jurisdiction? **(s2yq10)**
- ◻ **[1]** Yes
- ◻ **[0]** No
- ◻ **[2]** No, but we are working on developing one

11. *Optional:* Please provide any additional information about your health department's PrEP activities that you have not shared above or would like to expand on. In particular, NACCHO is interested in identifying successful strategies and practices for PrEP implementation. **(s2yq11text)**

Section IIIY: Next Steps for PrEP Implementation

Sections IIY, IIIY, IVY are for those who are currently engaged in PrEP in any manner (checked yes to s1q9)

1. Given current or realistic resources, what do you see as your health department's optimal role(s) as it relates to implementing PrEP in your jurisdiction? (Select all that apply) **(Variable values: unchecked=0, checked=1)**

- ☐ **(s3yq1_a)** Our optimal role is what we are currently doing to support PrEP implementation
- ☐ **(s3yq1_b)** Conducting community education and outreach
- ☐ **(s3yq1_c)** Conducting healthcare provider education and outreach
- ☐ **(s3yq1_d)** Conducting training events for health department staff
- ☐ **(s3yq1_e)** Convening or participating in a local or state working group on PrEP
- ☐ **(s3yq1_f)** Identifying local healthcare providers who are prescribing or willing to prescribe PrEP
- ☐ **(s3yq1_g)** Referring high-risk individuals to PrEP providers/programs
- ☐ **(s3yq1_h)** Delivering/prescribing PrEP via a health department clinic (e.g., STD clinic)
- ☐ **(s3yq1_i)** Collaborating with healthcare providers to support PrEP delivery (e.g., health department provides HIV/STD screening and/or adherence and behavioral risk-reduction counseling)
- ☐ **(s3yq1_j)** Funding community-based organizations and agencies to support PrEP implementation
- ☐ **(s3yq1_k)** Monitoring and evaluating PrEP uptake and impact
- ☐ **(s3yq1_l)** Participating in a demonstration site project/implementation pilot study
- ☐ **(s3yq1_m)** Other (please specify): ________**(s3yq1_mtext)**________

2. Of the questions below, which does your health department struggle with most as it considers the future of PrEP implementation? (Select up to two) **(Variable values: unchecked=0, checked=1)**

- ☐ **(s3yq2_a)** Is PrEP scalable to meet demand in our jurisdiction?
- ☐ **(s3yq2_b)** What is the health department's optimal role in supporting PrEP implementation?
- ☐ **(s3yq2_c)** Who would most benefit from PrEP in our jurisdiction?
- ☐ **(s3yq2_d)** How will PrEP be made accessible to those most in need?
- ☐ **(s3yq2_e)** How will we measure the uptake and impact of PrEP?
- ☐ **(s3yq2_f)** Other (please specify): ________**(s3yq2_ftext)**________
- ☐ **(s3yq2_g)** None of the above

3. Compared to where you are now, do you anticipate that your health department will seek to expand its level of engagement in PrEP implementation? **(s3yq3)**

- ☐ **[1]** Yes
- ☐ **[0]** No
- ☐ **[2]** Not sure

Section IVY: Resource and Assistance Needs

Sections IIY, IIIY, IVY are for those who are currently engaged in PrEP in any manner (checked yes to s1q9)

1. What resources or tools would be most helpful for advancing your health department's engagement in PrEP? (Select up to four resources or tools that apply) **(Variable values: unchecked=0, checked=1)**

- ☐ **(s4yq1_a)** Information about PrEP to share with healthcare providers
- ☐ **(s4yq1_b)** Education and outreach materials for community members
- ☐ **(s4yq1_c)** Protocols for PrEP referral from a health department clinic
- ☐ **(s4yq1_d)** Protocols for PrEP delivery via a health department clinic
- ☐ **(s4yq1_e)** Case studies/examples of health department PrEP implementation activities
- ☐ **(s4yq1_f)** Tools to assess healthcare provider interest in and knowledge of PrEP
- ☐ **(s4yq1_g)** Tools to assess community interest in and knowledge of PrEP
- ☐ **(s4yq1_h)** Tools to assess individual HIV risk/PrEP benefit
- ☐ **(s4yq1_i)** Information about third-party billing for PrEP
- ☐ **(s4yq1_j)** Additional funding for PrEP implementation → display question 1a
- ☐ **(s4yq1_k)** Guidance/direction from the state health department
- ☐ **(s4yq1_l)** Guidance or information on measuring PrEP uptake
- ☐ **(s4yq1_m)** Policy statements or position papers from national organizations (e.g., NACCHO)
- ☐ **(s4yq1_n)** Other (please specify): _______**(s4yq1_ntext)**_______

1a. What would you do with additional funding to support PrEP implementation? (Select all that apply) **(Variable values: unchecked=0, checked=1)**

- ☐ **(s4yq1a_a)** Plan for how to most effectively incorporate PrEP into HIV prevention education and services
- ☐ **(s4yq1a_b)** Develop educational materials about PrEP
- ☐ **(s4yq1a_c)** Communications activities related to PrEP
- ☐ **(s4yq1a_d)** Expand healthcare provider education efforts
- ☐ **(s4yq1a_e)** Develop and deliver HIV risk-reduction counseling and behavioral interventions for persons taking PrEP
- ☐ **(s4yq1a_f)** Evaluation activities for PrEP-related activities
- ☐ **(s4yq1a_g)** Program staff to conduct non-clinical PrEP-related activities (e.g., assessment of eligibility for insurance, navigation or linkage to providers)
- ☐ **(s4yq1a_h)** Personnel (e.g., healthcare providers) to prescribe PrEP and care for patients on PrEP
- ☐ **(s4yq1a_i)** Other (please specify): _______**(s4yq1a_itext)**_______

2. What type of assistance or training would be most helpful for advancing your health department's engagement in PrEP? (Select up to three) **(Variable values: unchecked=0, checked=1)**

- ☐ **(s4yq2_a)** In-person learning and training events
- ☐ **(s4yq2_b)** Web-based learning and training events
- ☐ **(s4yq2_c)** Direct technical or capacity building assistance
- ☐ **(s4yq2_d)** A community of practice, peer network, or other forum for health departments to discuss PrEP
- ☐ **(s4yq2_e)** Visiting a health department implementing PrEP
- ☐ **(s4yq2_f)** Other (please specify): _______**(s4yq2_ftext)**_______

3. What PrEP-related topics do you need to know more about? (Select up to three) **(Variable values: unchecked=0, checked=1)**

- ☐ **(s4yq3_a)** Identifying populations that might benefit from PrEP

- ☐ **(s4yq3_b)** Identifying individuals that might benefit from PrEP
- ☐ **(s4yq3_c)** Educating community members about PrEP
- ☐ **(s4yq3_d)** Educating healthcare providers about PrEP
- ☐ **(s4yq3_e)** Identifying healthcare providers prescribing or willing to prescribe PrEP
- ☐ **(s4yq3_f)** Medical costs and payment options for individuals on PrEP
- ☐ **(s4yq3_g)** Establishing a protocol for referral to a PrEP provider/program from a health department clinic
- ☐ **(s4yq3_h)** Monitoring and evaluating the uptake and impact of PrEP
- ☐ **(s4yq3_i)** Improving testing and prevention services for MSM
- ☐ **(s4yq3_j)** Other (please specify): _______**(s4yq3_jtext)**________

Public health or academic detailing is an education strategy in which professional clinical educators, trained health department staff, or trained academics visit medical practices to deliver information and provide resource kits with clinical information, tools for provider practice, and patient education materials to promote public health interventions in clinical practice. These services may be provided by trained health department staff or contractors.

4. Are you familiar with public health or academic detailing? **(s4yq4)**

- ☐ **[1]** Yes, I have heard of it before
- ☐ **[2]** Yes, my health department conducts or supports public health/academic detailing
- ☐ **[0]** No, I have not heard of this before

5. Is public health or academic detailing a strategy you would consider for increasing provider awareness and knowledge of PrEP? **(s4yq5)**

- ☐ **[1]** Yes, this is something the health department could consider doing.
- ☐ **[2]** Yes, this is something we are currently considering.
- ☐ **[3]** Yes, we currently have a public health detailing program that addresses PrEP.
- ☐ **[0]** No, (please specify why not): _______**(s4yq5text)**________

Section IIN: PrEP Awareness, Knowledge, and Interest

Sections IIN, IIIN, IVN are for those who are not currently engaged in PrEP in any manner (checked no to s1q9)

1. To what extent do you agree with the following statements:

	Strongly disagree	Disagree	Neither agree nor disagree	Agree	Strongly agree
Staff at your health department are knowledgeable about PrEP **(s2nq1_a)**	**[1]**	**[2]**	**[3]**	**[4]**	**[5]**
Leadership at your health department is supportive of incorporating PrEP into HIV prevention services/activities **(s2nq1_b)**	**[1]**	**[2]**	**[3]**	**[4]**	**[5]**
Given the incidence and prevalence of HIV in your jurisdiction, PrEP is an HIV prevention method that has the potential to make a significant impact on reducing new HIV infections **(s2nq1_c)**	**[1]**	**[2]**	**[3]**	**[4]**	**[5]**

2. Within the past 12 months, has your health department:

	Yes	No	Not yet, but we are planning to within the next 6 months	Don't know
Discussed (internally) how it might participate in supporting or providing PrEP? **(s2nq2_a)**	**[1]**	**[0]**	**[2]**	**[3]**
Discussed with HIV prevention partners in the community how PrEP might be incorporated into existing HIV prevention efforts? **(s2nq2_b)**	**[1]**	**[0]**	**[2]**	**[3]**
Discussed PrEP delivery with healthcare providers in the community? **(s2nq2_c)**	**[1]**	**[0]**	**[2]**	**[3]**
Met with or conducted assessments among community members or clinic patients to determine awareness, knowledge, and interest in PrEP? **(s2nq2_d)**	**[1]**	**[0]**	**[2]**	**[3]**

3. Have healthcare providers reached out to the health department with questions about PrEP? **(s2nq3)**

- ☐ **[1]** Yes
- ☐ **[0]** No
- ☐ **[2]** Don't know

4. Have community members reached out to the health department with questions about PrEP? **(s2nq4)**

- ☐ **[1]** Yes
- ☐ **[0]** No
- ☐ **[2]** Don't know

5. Who in your health department would be most likely to have a conversation about PrEP with a potential PrEP user? (Select up to three) **(Variable values: unchecked=0, checked=1)**

- ◻ **(s2nq5_a)** Sexual health/STD clinician
- ◻ **(s2nq5_b)** HIV clinician
- ◻ **(s2nq5_c)** Family planning practitioner
- ◻ **(s2nq5_d)** Disease Intervention Specialist (DIS)
- ◻ **(s2nq5_e)** Linkage to care specialist
- ◻ **(s2nq5_f)** HIV case manager
- ◻ **(s2nq5_g)** Outreach and education staff
- ◻ **(s2nq5_h)** Behavioral health specialist
- ◻ **(s2nq5_i)** Other (please specify): ________**(s2nq5_itext)**________
- ◻ **(s2nq5_j)** Not sure

6. Are there any community-based organizations or agencies in your jurisdiction that focus on HIV and/or STD prevention and education? **(s2nq6)**

- ◻ **[1]** Yes
- ◻ **[0]** No → skip question 6a
- ◻ **[2]** Don't know → skip question 6a

6a. Are any of these organizations or agencies engaged in PrEP implementation activities? **(s2nq6a)**

- ◻ **[1]** Yes
- ◻ **[0]** No
- ◻ **[2]** Don't know

7. Are there any healthcare providers in your community prescribing PrEP? **(s2nq7)**

- ◻ **[1]** Yes
- ◻ **[0]** No
- ◻ **[2]** Don't know

Section IIIN: Potential Future Engagement in PrEP Implementation

Sections IIN, IIIN, IVN are for those who not currently engaged in PrEP in any manner (checked no to s1q9)

The questions in this section focus on what *you think your health department could potentially do to support PrEP implementation*. This information will help identify the most likely roles for local health departments in PrEP implementation and inform efforts to support health department decision-making about incorporating PrEP into existing HIV prevention education and services.

1. Please select the activities you think your health department could do or would be interested in doing to support PrEP implementation. (Select all that apply) **(Variable values: unchecked=0, checked=1)**

- ☐ **(s3nq1_a)** Conducting community education and outreach
- ☐ **(s3nq1_b)** Conducting healthcare provider education and outreach
- ☐ **(s3nq1_c)** Conducting training events for health department staff
- ☐ **(s3nq1_d)** Participating in PrEP planning conversations with local/regional HIV prevention partners
- ☐ **(s3nq1_e)** Convening or participating in a local or state working group on PrEP
- ☐ **(s3nq1_f)** Developing a list of local healthcare providers prescribing or willing to prescribe PrEP
- ☐ **(s3nq1_g)** Referring high-risk individuals to PrEP providers/programs
- ☐ **(s3nq1_h)** Delivering/prescribing PrEP via a health department clinic (e.g., STD clinic)
- ☐ **(s3nq1_i)** Collaborating with healthcare providers to support PrEP delivery (e.g., health department provides HIV/STD screening and/or adherence and behavioral risk-reduction counseling; provider prescribes PrEP and conducts routine lab tests)
- ☐ **(s3nq1_j)** Funding community-based organizations and agencies to support PrEP implementation
- ☐ **(s3nq1_k)** Monitoring and evaluating PrEP uptake and impact
- ☐ **(s3nq1_l)** Participating in a demonstration site project/implementation pilot study
- ☐ **(s3nq1_m)** Other (please specify): ________**(s3nq1_mtext)**________
- ☐ **(s3nq1_n)** None of the above

2. What challenges has your health department faced that have prevented it from engaging in PrEP, or what challenges do you anticipate the health department would face to engagement in PrEP? (Select all that apply) **(Variable values: unchecked=0, checked=1)**

- ☐ **(s3nq2_a)** Lack of PrEP awareness and knowledge among health department staff
- ☐ **(s3nq2_b)** Lack of support or interest from health department leadership
- ☐ **(s3nq2_c)** Uncertainty about PrEP as an effective HIV prevention strategy (e.g., belief that the benefits are outweighed by possible behavioral disinhibition and increased STDs, or that PrEP is a party drug)
- ☐ **(s3nq2_d)** Limited staff capacity to support PrEP implementation activities
- ☐ **(s3nq2_e)** Lack of enough healthcare providers willing to provide PrEP
- ☐ **(s3nq2_f)** Concern about financial access to PrEP (for individuals interested in taking PrEP)
- ☐ **(s3nq2_g)** Concern about inadequate reimbursement from third-party payers for PrEP-related services
- ☐ **(s3nq2_h)** Other (please specify): ________**(s3nq2_htext)**________
- ☐ **(s3nq2_i)** Health department is not sure what it should or could be doing
- ☐ **(s3nq2_j)** PrEP has not been an area of focus for our health department, so we have not faced any significant challenges to date

3. Of the questions below, which question do you think your health department would struggle with the most if it were to begin considering how to support PrEP implementation? (Select up to two) **(Variable values: unchecked=0, checked=1)**

- ☐ **(s3nq3_a)** Is PrEP scalable to meet demand in our jurisdiction?

- ☐ **(s3nq3_b)** What is the health department's optimal role in supporting PrEP implementation?
- ☐ **(s3nq3_c)** Who would most benefit from PrEP in our jurisdiction?
- ☐ **(s3nq3_d)** How will PrEP be made accessible to those most in need?
- ☐ **(s3nq3_e)** How will we measure the uptake and impact of PrEP?
- ☐ **(s3nq3_f)** Don't know
- ☐ **(s3nq3_g)** Other (please specify): _______**(s3nq3_gtext)**________

Section IVN: Resource and Assistance Needs

Sections IIN, IIIN, IVN are for those who are not currently engaged in PrEP in any manner (checked no to s1q9)

> The questions in this section are intended to help identify what resources and assistance your health department might benefit from, *if you were to begin considering how to incorporate PrEP into prevention education and services.*

1. What resources or tools would be most helpful for advancing your health department's engagement in PrEP? (Select up to four resources or tools that apply) **(Variable values: unchecked=0, checked=1)**

- ☐ **(s4nq1_a)** Information about PrEP for health department staff
- ☐ **(s4nq1_b)** Information about PrEP to share with healthcare providers
- ☐ **(s4nq1_c)** Education and outreach materials for community members
- ☐ **(s4nq1_d)** Protocols for PrEP referral from a health department clinic
- ☐ **(s4nq1_e)** Protocols for PrEP delivery via a health department clinic
- ☐ **(s4nq1_f)** Case studies/examples of health department PrEP implementation activities
- ☐ **(s4nq1_g)** Tools to assess healthcare provider interest in and knowledge of PrEP
- ☐ **(s4nq1_h)** Tools to assess community interest in and knowledge of PrEP
- ☐ **(s4nq1_i)** Tools to assess individual HIV risk/PrEP benefit
- ☐ **(s4nq1_j)** Information about third-party billing for PrEP
- ☐ **(s4nq1_k)** Funding for PrEP implementation → Display question 1a
- ☐ **(s4nq1_l)** Guidance/direction from the state health department
- ☐ **(s4nq1_m)** Guidance or information on measuring PrEP uptake
- ☐ **(s4nq1_n)** Policy statements or position papers from national organizations (e.g., NACCHO)
- ☐ **(s4nq1_o)** Other (please specify): ________**(s4nq1_otext)**________

1a. What would you do with funding to support PrEP implementation? (Select all that apply) **(Variable values: unchecked=0, checked=1)**

- ☐ **(s4nq1a_a)** Plan for how to most effectively incorporate PrEP into HIV prevention education and services
- ☐ **(s4nq1a_b)** Develop educational materials about PrEP
- ☐ **(s4nq1a_c)** Communications activities related to PrEP
- ☐ **(s4nq1a_d)** Expand healthcare provider education efforts
- ☐ **(s4nq1a_e)** Develop and deliver HIV risk-reduction counseling and behavioral interventions for persons taking PrEP
- ☐ **(s4nq1a_f)** Evaluation activities for PrEP-related activities
- ☐ **(s4nq1a_g)** Program staff to conduct non-clinical PrEP-related activities (e.g., assessment of eligibility for insurance, navigation or linkage to providers)
- ☐ **(s4nq1a_h)** Personnel (e.g., healthcare providers) to prescribe PrEP and care for patients on PrEP
- ☐ **(s4nq1a_i)** Other (please specify): ________**(s4nq1a_itext)**________

2. What type of assistance or support would be most helpful for advancing your health department's engagement in PrEP? (Select up to three) **(Variable values: unchecked=0, checked=1)**

- ☐ **(s4nq2_a)** In-person learning and training events
- ☐ **(s4nq2_b)** Web-based learning and training events
- ☐ **(s4nq2_c)** Direct technical or capacity building assistance
- ☐ **(s4nq2_d)** A community of practice, peer network, or other forum for health departments to discuss PrEP

- ☐ **(s4nq2_e)** Visiting a health department implementing PrEP
- ☐ **(s4nq2_f)** Other (please specify): _______**(s4nq2_ftext)**________

3. What PrEP-related topics would you need to know more about? (Select up to three) **(Variable values: unchecked=0, checked=1)**

- ☐ **(s4nq3_a)** Identifying populations that might benefit from PrEP
- ☐ **(s4nq3_b)** Identifying individuals that might benefit from PrEP
- ☐ **(s4nq3_c)** Educating community members about PrEP
- ☐ **(s4nq3_d)** Educating healthcare providers about PrEP
- ☐ **(s4nq3_e)** Identifying healthcare providers prescribing or willing to prescribe PrEP
- ☐ **(s4nq3_f)** Medical costs and payment options for individuals on PrEP
- ☐ **(s4nq3_g)** Establishing a protocol for referral to a PrEP provider/program from a health department clinic
- ☐ **(s4nq3_h)** Monitoring and evaluating the uptake and impact of PrEP
- ☐ **(s4nq3_i)** Improving testing and prevention services for MSM
- ☐ **(s4nq3_j)** Other (please specify): _______**(s4nq3_jtext)**________

4. Do you anticipate that your health department will become engaged in PrEP implementation activities in the future? **(s4nq4)**

- ☐ **[1]** Yes, it is likely over the next 12 months
- ☐ **[2]** Yes, it is likely over the next 1-2 years
- ☐ **[3]** Yes, it is likely over the next 2-4 years
- ☐ **[4]** Undecided
- ☐ **[0]** No, it is unlikely that the health department will incorporate PrEP into its HIV prevention and education services and activities

Section V: Concluding Questions

1. Before submitting your responses, is there anything else you would like to share or go into greater detail about? If so, please use the space below to do so. **(s5q1text)**
